# Assessing DNA Barcoding as a Tool for Species Identification and Data Quality Control

**DOI:** 10.1371/journal.pone.0057125

**Published:** 2013-02-19

**Authors:** Yong-Yi Shen, Xiao Chen, Robert W. Murphy

**Affiliations:** 1 School of Life Sciences, Xiamen University, Xiamen, People's Republic of China; 2 Zhejiang Mariculture Research Institute, Wenzhou, People's Republic of China; 3 State Key Laboratory of Genetic Resources and Evolution, Kunming Institute of Zoology, the Chinese Academy of Sciences, Kunming, People's Republic of China; 4 Centre for Biodiversity and Conservation Biology, Royal Ontario Museum, Toronto, Canada; Biodiversity Insitute of Ontario - University of Guelph, Canada

## Abstract

In recent years, the number of sequences of diverse species submitted to GenBank has grown explosively and not infrequently the data contain errors. This problem is extensively recognized but not for invalid or incorrectly identified species, sample mixed-up, and contamination. DNA barcoding is a powerful tool for identifying and confirming species and one very important application involves forensics. In this study, we use DNA barcoding to detect erroneous sequences in GenBank by evaluating deep intraspecific and shallow interspecific divergences to discover possible taxonomic problems and other sources of error. We use the mitochondrial DNA gene encoding cytochrome *b* (*Cytb*) from turtles to test the utility of barcoding for pinpointing potential errors. This gene is widely used in phylogenetic studies of the speciose group. Intraspecific variation is usually less than 2.0% and in most cases it is less than 1.0%. In comparison, most species differ by more than 10.0% in our dataset. Overlapping intra- and interspecific percentages of variation mainly involve problematic identifications of species and outdated taxonomies. Further, we detect identical problems in *Cytb* from Insectivora and Chiroptera. Upon applying this strategy to 47,524 mammalian *CoxI* sequences, we resolve a suite of potentially problematic sequences. Our study reveals that erroneous sequences are not rare in GenBank and that the DNA barcoding can serve to confirm sequencing accuracy and discover problems such as misidentified species, inaccurate taxonomies, contamination, and potential errors in sequencing.

## Introduction

Publically available, GenBank (http://www.ncbi.nlm.nih.gov/sites/entrez) provides an annotated suite of open access, nucleotide sequences and, when applicable, their amino acid translations. GenBank relies on direct submissions from individual laboratories. Because of increasing efficiencies of sequencing and molecular research, the volume of data is explosively increasing. The sheer volume of new information necessarily translates into the accumulation of errors. For example, more than half of all published human mtDNA studies have errors [1] and 5.0% error in mitochondrial 16S rRNA sequence data occurs in public repositories [2]. Although attention focuses on the quality of the human mtDNA database [3]–[5], little effort focuses on the extent of erroneous sequences arising from the misidentification of species, sampling error, and contamination, especially in phylogenetic analyses. Unfortunately, the 'garbage in, garbage out' rule applies. If the data are not reliable, forensic analyses will have limited repeatability, phylogenies will introduce confusion, and in both cases errors may even lead to irreproducible results.

DNA barcoding usually consists of a fragment of the mitochondrial gene cytochrome oxidase c subunit I (*Cox1, mt-co1, COI*) but other genes are also employed, sometimes with varying levels of success [6], [7]. The method has many applications among which it is an efficient means of identifying species because levels of divergence among individuals are usually much lower of the same species than between closely related species [8]–[14]. Barcoding successfully identifies a great diversity of species [15]–[27]. A sequence from a misidentified species will result in a high level of intraspecific K2P divergence [28]. In this study, we use divergence values to detect potential errors in sequences in GenBank to assess and improve the quality of the data.

Phylogenetic/genealogical analyses commonly use cytochrome *b* (*Cytb*) sequences. Thus, we use a dataset of 2555 *Cytb* sequences of turtles to test the power of DNA barcoding to confirm species identities and pinpoint problems. If this approach proves to be a powerful means of identifying errors, we can expect it to detect potential flaws in other groups. Thus, we further analyze 3516 and 6269 *Cytb* sequences in the Insectivora and Chiroptera. *CoxI* is the most widely used marker for DNA barcoding and, therefore, we also analyze 47,524 mammalian *CoxI* sequences in GenBank.

## Results and Discussion

**Figure 1:**
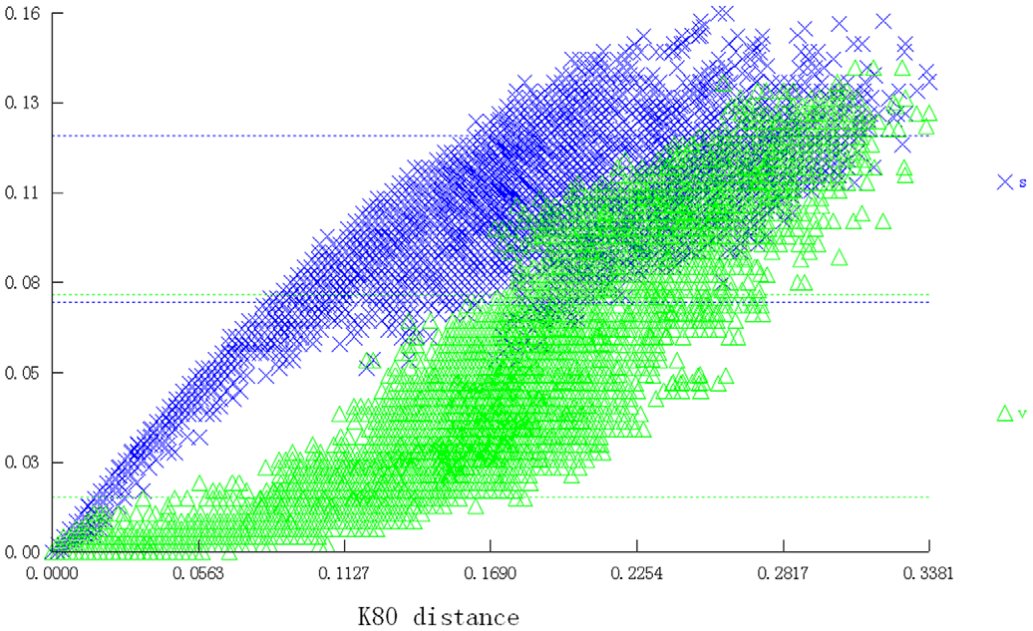
Transitions and transversions plotted against the pairwise sequence divergence for turtles using 924bp of the *Cytb* DNA barcode. All three codon positions are used.

The compiled dataset of *Cytb* sequences from turtles was used to evaluate the ability of DNA barcoding to detect erroneous sequences in GenBank. The lengths of available *Cytb* sequences vary, and consequently a clear tradeoff exists between maximizing the length of the alignments and taxonomic coverage. The final data set consists of 1686 fragments of 924bp. When we set *Cytb* GenBank accession NC_015986 as the standard for all comparisons, the available fragments ranged from 75bp to 998bp. Given that the goal is to identify erroneous species and data, we use neighbor joining (NJ) trees as an efficient means of summarizing divergence between the sequences. Not surprising, the topology of the NJ phenogram is almost identical to trees obtained using morphology [29], nuclear genes [30]–[32], and mitochondrial genes [31], although the bootstrap values are smaller, as expected, and some branching orders remain unsolved (Figure S1). Nucleotide diversity averages 16.0% and transitions are saturated at about 15.0% when all codon positions are compared (Figure 1).

**Figure 2:**
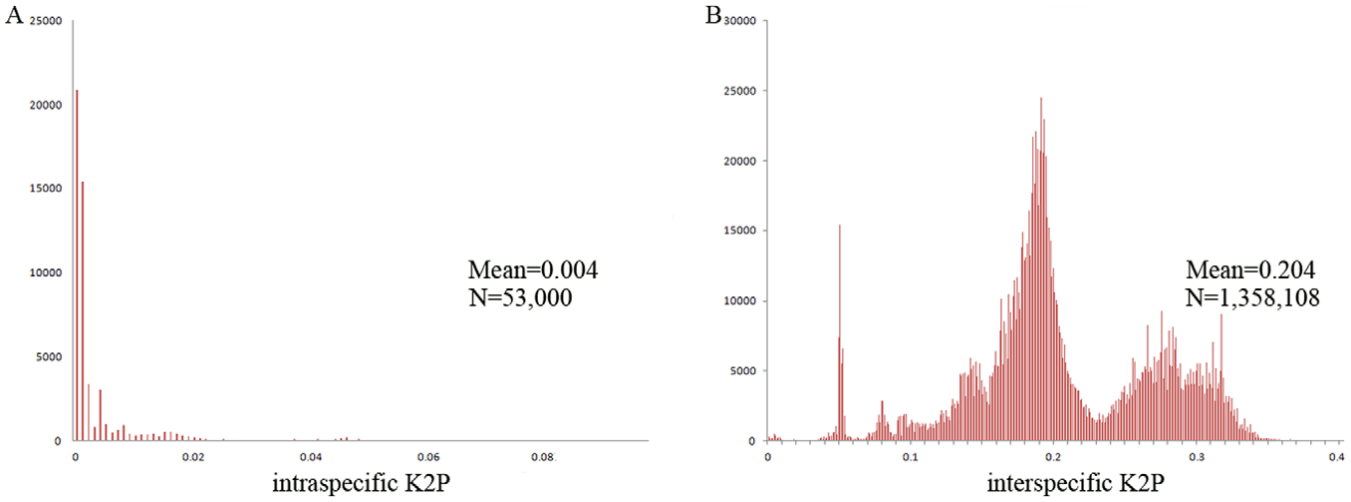
Intra- (A) and interspecific (B) pairwise divergences (Kimura 2-parameter). Majority of intraspecific divergences are less than 5.0% (A); majority of interspecific divergences exceed 8.0% (B).

To identify species, we assume that the intraspecific differences are much less than interspecific divergences. Intraspecific divergences rarely exceed 5.0% and most are less than 1.0% in this dataset. In contrast, interspecific divergences usually exceed 8.0% (Figure 2). However, some notable exceptions to the pattern occur. *Ocadia glyphistoma* (AY434596) hardly differs from *Mauremys annamensis* (0.5–4.9%), and *M. pritchardi* is very similar to *M. mutica* (0.0%–6.4%). These divergence values are substantially lower than most interspecific values. This finding conforms to previous studies [33], [34]. The K2P divergence between *Mauremys megalocephala* and *M. reevesi* is 0.0–1.0% and such low values imply conspecificity. *Cuora trifasciata* (AY434627) has a very close relationship with *Cuora aurocapitata* (0.3%–2.5%) and *Cuora pani* (0.3–1.9%). Thus, *Cuora aurocapitata*, *Curora pani*, and *Curora zhoui* appear to have recent origins [33]–[37]. Similarly, the intraspecific divergences for *Pseudemys nelsoni* (0.0%), *Pseudemys rubriventris* (0.2–0.5%), *Pseudemys suwanniensis* (0.1%), *Pseudemys peninsularis* (0.0–0.4%), *Pseudemys texana* (0.1%), *Pseudemys gorzugi* (0.0%), *Pseudemys alabamensis* (0.0–0.5%), *Pseudemys concinna* (0.0–0.7%) overlap with their interspecific divergences (0.0–1.1%). In genus *Graptemys*, the intraspecific divergence is 0.0–0.3%, while the interspecific divergence in this genus is 0.0–2.8%. These results suggest that the species in these two genera may have recent origins.

*Ocadia philippeni* and *Mauremys iversoni* show relatively low divergence (0.3–0.5%). Other confusion exists. One sequence of *Pangshura smithii* (AM495294) clusters with *Pangshura tentoria* (0.0–0.7%). Further, one sequence of *Pangshura tentoria* (AM495328) clusters with *Pangshura smithii* (0.0–0.2%).

*Cyclemys tcheponensis* (AY434577), *Cyclemys shanensis* (AJ604513), and *Cyclemys dentate* (AY434579) show shallow interspecific divergence from *Cyclemys oldhamii* (0.0–1.7%). This unexpected finding implies either that these “species” are, in fact, conspecific or the source specimens are not correctly identified. The same problem occurs for several pairs of taxa. *Pelusios chapini* (FR716922) is similar to *Pelusios castaneus* (1.3–1.9%). *Phrynops geoffroanus* (JX139069) and *Mesoclemmys gibba* (JX139068) have the same sequence. Two sequences of *Cuora picturata* (NC_017878 and JF712890) are the same as those of *Cuora bourreti* (NC_017885 and JN020145). *Sternotherus odoratus* (GQ896189) is identical to sequences of *Sternotherus carinatus. Cyclemys atripons* (NC_010970 and AY434617) has shallow interspecific divergence (0.0–0.9%) from *Cyclemys pulchristriata*. In these cases, the lack of difference between the species indicates the need for further study. Either corrections are required in GenBank or the taxonomy of the taxa needs reexamination.

Deep intraspecific divergence also occurs in the database. For example, *Cuora flavomarginata* has two divergent clusters (EU708434 and NC_012054 vs. AY434570, GQ896188, and AY434606) that differ by 6.5–7.4%. Two sequences of *Deirochelys reticularia* (FJ770592-93 vs. HE590299) differ by 19.0%. *Orlitia borneensis* (AY434619 vs. AJ564464) differ by 12.4%. One sequence of *Palea steindachneri* (AY743417) deeply diverges from other sequences (AY259552 and NC_013841) (15.4–16.0%). *Pelochelys cantorii* (JF719809) has intraspecific divergence of 19.1–19.2%. These cases also suggest either misidentifications or problematic taxonomies.

The problematic sequence for *Palea steindachneri* (AY743417) is quite similar to that of *Pelochelys cantorii* (JF719809; 1.6%). *Sacalia quadriocellata* (NC_011819) shows shallow interspecific divergence with *Sacalia bealei* (0.2–1.1%) yet deep intraspecific variation 9.3–10.0%. Thus, the specimens for sequences AY743417 and NC_011819 may be misidentified. Such contradictory values document that to err is human, and yet DNA barcoding can detect such errors.

No particular level of divergence can serve to identify species. Rather, such data can point to taxa that need additional study. K2P distances between *Rhinoclemmys diademata*, *R. punctularia*, and *R. melanosterna* range from 1.4% to 2.3%. The low levels of divergence indicate either recent divergences or perhaps a taxon-specific slowing of the molecular clock. More importantly, only one sequence is available for each species and the result indicates a need for further study using more samples. Similarly, newly described *Emys trinacris* [38] forms an independent lineage that is the sister group of *E. orbicularis*. However, interspecific divergences are very low (0.7–2.3%) and intraspecific divergences of *E. orbicularis* range from 0.0 to 2.0%.

Many currently recognized taxonomic names are composites of cryptic species complexes [39]. *Testudo graeca* (six subspecies) and *Geochelone pardalis* (two subspecies) have complex relationships. Intraspecific divergence in the former species ranges from 0.0 to 8.1% and in the latter from 0.0 to 12.4%. Thus, these two species complexes require further attention as they may be polytypic. DNA barcoding has accelerated the rates of taxonomic discovery and descriptions to meet or exceed rates of biodiversity loss [40]–[42]. In contrast to great variation, 16 samples of *Indotestudo forstenii* share one haplotype. This endangered species has a critically low level of diversity necessitating that greater attention must be paid to its conservation status.

Overlapping intra- and interspecific levels of divergences indicate not only natural variation but also potential errors in GenBank and taxonomic conundrums. Among the several new species of turtles described during the last 20 years based on morphology, most were controversial. Our study affirms that DNA barcodes can provide critical data before the description of a new species, and this may involve forensics into geographic origins [43].

To test if our barcoding strategy is applicable to other taxa, we analyzed two orders of mammals, shrews (Insectivora) and bats (Chiroptera). Both groups contain a large number of species and species identity can be confusing. Identical to turtles, analyses detect potential errors in GenBank sequences, as well as taxonomic uncertainties (Table 1).

**Table 1:** Potential errors for *cytb* sequences in Insectivora and Chiroptera.

Potential error sequences	reasons	
DQ869420 *Artibeus planirostris;* DQ869421 *Artibeus planirostris;* DQ869419 *Artibeus planirostris*		Shallow interspecific divergence with *Artibeus jamaicensis* (0.1%–5.0%) but deep intraspecific divergence (6.0%–7.9%)
U66502*;* AY144339*;* AY144338 *Artibeus intermedius*		Shallow divergence with *Artibeus lituratus* (1.1%–3.8%)
AY572353 *Artibeus jamaicensis;* AY572355 *Artibeus jamaicensis*		Shallow interspecific divergence with *Artibeus schwartzi* (0.4%–1.3%) but deep intraspecific divergence (4.2%–6.9%)
DQ985486 *Artibeus jamaicensis;* U66504 *Artibeus jamaicensis;* U66503 *Artibeus jamaicensis*		Shallow interspecific divergence with *Artibeus planirostris* (0.3%–4.5%) but deep intraspecific divergence (4.9%–8.8%)
DQ869386*;* U66513*;* U66512*;* U66516 *Artibeus glaucus*		Deep intraspecific divergence (7.4%–13.2%)
DQ077405 *Micronycteris minuta;* AY380753 *Micronycteris schmidto*		Shallow interspecific divergence (0.4%)
AY380756 *Micronycteris microtis*		Shallow interspecific divergence with *Micronycteris megaloti* (0.4%–3.5%) but deep intraspecific divergence (5.8%)
AB085735 *Miniopterus fuliginous*		Shallow divergence with *Miniopterus schreibers* (0.4%–1.4%)
EF570882 *Plecotus auritus*		Deep intraspecific divergence (11.7%)
EF517305 *Miniopterus magnater*		Shallow divergence with *Miniopterus schreibers* (1.0%–1.7%)
AB085738 *Vespertilio superans*		Shallow divergence with *Vespertilio sinensis* (0.7%–1.2%)
AB106605 *Myotis mystacinus*		Deep intraspecific divergence (20.3%–20.6%)
AY665142*;* AY665145*;* AY665161*;* AY665164 *Myotis aurascens*		Deep intraspecific divergence (8.6%–19.9%)
AY324470–AY324473 *Apomys insignis;* AY324467–AY324469 *Apomys hylocoetes*		Show shallow interspecific divergence (0.0–0.4%)
AB077073 *Crocidura dsinezumi*		Shallow interspecific divergence with *Crocidura lasiura* (1.2%) but deep intraspecific divergence (7.1%–8.5%)
AY994386 Crocidura suaveolens		Shallow interspecific divergence with *Crocidura sibirica* (0.2%–0.7%) but deep intraspecific divergence (8.5%)
AY994373 *Crocidura gueldenstaed*		Shallow divergence with *Crocidura sibirica* (0.3%–0.6%)
AY926383 *Dipodomys merriami;* AY926371 *Dipodomys insularis;* AY926370 *Dipodomys margaritae*		Shallow interspecific divergence (1.0%–1.1%)
AB175092–AB175094 *Chimarrogale himalayic;* AB107874–AB107875 *Chimarrogale himalayic*		Deep intraspecific divergence (14.3%–14.8%)
AB175114–AB175115 *Episoriculus caudatus;* AB175112–AB175113 *Episoriculus caudutus*		Deep intraspecific divergence (13%–13.4%)
AY014927–AY014930, EU088307 *Sorex ugyunak;* AY014916–AY014920 *Sorex camtschatica;* AY014931–AY014933 *Sorex hydrodromus;* AY014921 *Sorex portenkoi;* AY014922–AY014926 *Sorex jacksoni*		Shallow interspecific divergence (0.0–0.8%)
AY014934–AY014935 *Sorex preblei;* AY014938–AY014940 *Sorex haydeni*		Shallow interspecific divergence (0.0–0.9%)

*CoxI* is the most widely used marker for DNA barcoding. Therefore, we also analyze 47,524 mammalian *CoxI* sequences in GenBank. Not surprising, many potential errors occur (Table S1). This result suggests that the paradox of deep intraspecific and shallow interspecific K2P distances can detect potential errors. This paradox is likely to be useful for a variety of popular genes such as 12S and 16S. If we exclude human sequences, primates have the highest error ratio (2.12%). When we do not exclude human sequences, even-toed ungulates have the highest error ratio (1.68%), as Table S2 shows.

In view of an explosive amount of data deposited in GenBank from an increasing number of laboratories, our study shows that erroneous sequences are not rare. In addition to artificial technological errors in sequencing, sample mix-up, contamination, and incorrect species identification constitute other possible sources of error. Erroneous data may strongly impact critical forensic applications, and result in confused taxonomies and phylogenies. Such errors are often hard to detect, and all too frequently there is no confirmation of either taxonomic accuracy or the possibility of contamination. The paradox of deep intraspecific and shallow K2P interspecific differences suggest that further verification of accuracy is necessary. Certainly, not all paradoxes owe to contamination and inaccurate identifications of species. Problematic and outdated taxonomies are also involved. Once reliable data are available for each species, and especially from type localities, it is possible to easily determine the source of the problematic sequences, be that sequencing errors or invalid taxonomies. The global initiative to DNA barcode all species of amphibians and reptiles – Cold Code [44] -- seeks to suggest corrections to GenBank. Thus, DNA barcoding is not only valuable for identifying species, but it can play an important role in detecting potential errors in GenBank.

## Materials and Methods

### 3.1 Source of data

We used the query “((cytb[gene] or "cytochrome b"[gene]) AND "vertebrates"[porgn:__txid7742]) AND "turtles"[porgn:__txid8459] AND 100:20000[SLEN]” to search for *Cytb* sequences of turtles in NCBI. Similarly, we used the query “((((cytb[gene] or "cytochrome b"[gene]) AND "vertebrates"[porgn:__txid7742]) AND 100:20000[SLEN]) AND "mammals"[porgn:__txid40674]) AND "bats"[porgn:__txid9397]” for bats, and “((((cytb[gene] or "cytochrome b"[gene]) AND "vertebrates"[porgn:__txid7742]) AND 100:20000[SLEN]) AND "mammals"[porgn:__txid40674]) AND "insectivores"[porgn:__txid9362]” for insectivores. For *CoxI*, we queried “((Cox1[gene] or "cytochrome c oxidase subunit I"[gene] or CoxI[gene] or COI[gene]) AND "vertebrates"[porgn:__txid7742]) AND "mammals"[porgn:__txid40674] AND 100:20000[SLEN]” to search for all mammalian *CoxI* sequences. In total, 2555 mitochondrial *Cytb* sequences of turtles, and 3516 and 6269 *Cytb* sequences of Insectivora and Chiroptera, respectively, were downloaded from GenBank. Additionally, 47,524 *Cox1* sequences for mammals were obtained from GenBank on 1 September 2012.

### 3.2 Data Analysis

The datasets for *Cytb* and *CoxI* were treated independently. All datasets were firstly aligned by MAFFT – a fast multiple sequence alignment program [45]. The alignments were trimmed by deleting the flanking regions of *Cytb* and *CoxI*. The trimmed sequences were aligned again by Clustal ×1.8 [46] to obtain more accurate alignments. These alignments were examined by eye and when required adjusted to exclude obvious alignment errors. The length of these published sequences varied. To obtain the maximum amount of homologous sequences. Accordingly, we obtained a final dataset that sought the greatest taxonomic diversity while considering the longest sequences by deleting outliers. All the datasets were available upon request.

For each dataset, A neighbor-joining tree the distance was created to provide a graphic representation of the patterning of divergences among species [47]. Sequence divergences were estimated using the K2P distance model [28] in MEGA 4 [48]. Sequences that had deep intraspecific or shallow interspecific K2P divergences were recorded as being potential errors. Then, we further checked their nucleotide sequences and its phylogenetic position by eye.

Transition saturation was tested by plotting the estimated number of transitions and transversions against genetic divergence using DAMBE [49]. Third codon positions and the first two codon positions were tested separately and combined.

## Supporting Information

Figure S1 **Neighbor-joining tree using 924 bp** ***Cytb*** **sequences for turtles.**
(TIF)

Table S1 **Potential error** ***CoxI*** **sequences in mammals.**
(DOC)

Table S2 **Potential error ratio for** ***CoxI*** **sequences in mammals.**
(DOCX)
