# Supplementary material for: Assessing DNA Barcoding as a Tool for Species Identification and Data Quality Control
Source: PLoS One. 2013 Feb 19;8(2):e57125. doi: 10.1371/journal.pone.0057125 (PMC3576373; doi:10.1371/journal.pone.0057125)
Supplement: Table S1 — Potential error CoxI sequences in mammals. (DOC) [file pone.0057125.s002.doc]

| Potential error sequences | reasons |
| --- | --- |
| JF342908 *Canis lupus* | Shallow interspecific divergence with *Canis latrans* |
| EU835716 *Alces alces* | Shallow interspecific divergence with *Rangifer tarandus* |
| JF443316- JF443322 *Odocoileus hemionus* | Shallow interspecific divergence with *Odocoileus virginianus* |
| JN632595 *Alces alce* | Shallow interspecific divergence with *Alces americanus* |
| JN632660 *Mazama nemorivaga* | Shallow interspecific divergence with *Mazama gouazoupira* |
| HM204518 *Cervus elaphus*  HM204517 *Cervus eldi*  HM204514-HM204516,GQ329016-GQ329017, EF035448, NC_008414 *Rusa unicolor*  HM049636 *Cervus albirostris*  NC_016707 *Przewalskium albirostris*  HM204510 *Cervus timorensis* | Shallow interspecific divergence with *Cervus nippon* |
| JN632626 *Connochaetes gnou* | Shallow interspecific divergence with *Connochaetes taurinus* |
| HQ644111, JN632621 *Cephalophus rufilatus* | Shallow interspecific divergence with *Cephalophus nigrifrons* |
| JN632591*Addax nasomaculatus* | Shallow interspecific divergence with *Oryx dammah* |
| JN632604 *Bos gaurus*  HQ269429 *Bos frontalis* | Shallow interspecific divergence with each other |
| *Bos taurus*, *Bos javanicus*, *Bos indicus*, *Bos primigenius* | Shallow interspecific divergence with each other |
| DQ534055, GU117615 *Lama pacos* | Shallow interspecific divergence with *Vicugna vicugna* |
| AP003426, NC_012102, GU117612 *Lama glama* | Shallow interspecific divergence with *Lama pacos* |
| AJ277029 *Physeter macrocephalus* | Shallow interspecific divergence with *Physeter catodon* |
| JF442786 *Mesoplodon stejnegeri* | Shallow interspecific divergence with *Ziphius cavirostris* |
| EF568613*Ziphius cavirostris*  EU496313 *Mesoplodon europaeus* | Shallow interspecific divergence with each other |
| JF442782 *Delphinus delphis* | Shallow interspecific divergence with *Phocoenoides dalli* |
| JF442789 *Phocoena phocoena* | Shallow interspecific divergence with *Delphinapterus leucas* |
| *Delphinus capensis*  *Delphinus delphis* | Shallow interspecific divergence with each other |
| EF090643*Stenella coeruleoalba* | Shallow interspecific divergence with *Delphinus delphis* |
| EF568714 *Stenella frontalis* | Shallow interspecific divergence with *Stenella clymene* |
| EU496342 *Stenella coeruleoalba* | Shallow interspecific divergence with *Stenella clymene* |
| JF339973*Globicephala melas* | Shallow interspecific divergence with *Orcinus orca* |
| AB547250 *Bos taurus* | Deep intraspecific divergence |
| EF636539 *Sorex granarius* | Shallow interspecific divergence with *Sorex araneus* |
| GU981242 *Episoriculus macrurus* | Shallow interspecific divergence with *Episoriculus caudatus* |
| GU981225 *Crocidura fuliginosa*  JF444469 *Suncus murinus* | Shallow interspecific divergence with each other |
| JF436025 *Sorex caecutiens* | Shallow interspecific divergence with *Sorex cinereus* |
| NC_006516 *Metachirus nudicaudatus* | Shallow interspecific divergence with *Didelphis marsupialis* |
| JF912199, NC_015529 *Mammuthus columbi* | Shallow interspecific divergence with *Mammuthus primigenius* |
| JF444474 *Tapirus terrestris*  JF444473 *Tapirus indicus* | Shallow interspecific divergence with each other |
| JF445287 *Rattus tanezumi* | Shallow interspecific divergence with *Rattus andamanensis* |
| JF445260 *Rattus rattus* | Shallow interspecific divergence with *Rattus andamanensis* |
| FR775808, FR775814, FR775818, FR775809 *Rattus tiomanicus* | Shallow interspecific divergence with *Rattus andamanensis* |
| JF459864, JF459865 *Rattus rattus*  HM217522, EF186628, EF186629 *Rattus tiomanicus* | Shallow interspecific divergence with each other |
| EF156861, EU107500 *Chaetodipus intermedius*  JF444291, JF444292 *Chaetodipus penicillatus* | Shallow interspecific divergence with each other |
| JQ601445 *Tamias minimus* | Shallow interspecific divergence with *Tamias striatus* |
| JQ601192 *Proechimys cuvieri* | Shallow interspecific divergence with *Proechimys guyannensis* |
| JQ601191 *Proechimys guyannensis* | Shallow interspecific divergence with *Proechimys cuvieri* |
| JQ601186 *Proechimys guyannensis* | Shallow interspecific divergence with *Hylaeamys megacephalus* |
| JQ601228 *Proechimys cuvieri* | Shallow interspecific divergence with *Hylaeamys megacephalus* |
| JF492254, JF492170 *Handleyomys rostratus* | Shallow interspecific divergence with *Handleyomys alfaroi* |
| JF446230 *Reithrodontomys gracilis* | Shallow interspecific divergence with *Ototylomys phyllotis* |
| JF446208, JF446209, JF446191 *Reithrodontomys gracilis* | Shallow interspecific divergence with *Reithrodontomys spectabilis* |
| JQ600683-JQ600687 *Peromyscus nudipes*  JQ600989-JQ600991, JQ600987 *Peromyscus gymnotis* | Shallow interspecific divergence with each other |
| HM165298 *Neodon irene* | Shallow interspecific divergence with *Microtus limnophilus* |
| DQ0155676 *Microtus rossiaemeridionalis*  NC_008064 *Microtus levis* | Shallow interspecific divergence with each other |
| HM165290 *Eothenomys miletus* | Shallow interspecific divergence with *Eothenomys custos* |
| HM165312 *Eothenomys cachinus*  HM165316 *Eothenomys eleusis* | Shallow interspecific divergence with each other |
| JF499324 *Myodes glareolus* | Shallow interspecific divergence with *Myodes rutilus* |
| NC_012461 *Rattus praetor*  EF186569 *Rattus novaeguineae* | Shallow interspecific divergence with each other |
| JF459710 *Maxomys ochraceiventer* | Shallow interspecific divergence with *Maxomys rajah* |
| JQ667986 *Praomys rostratus* | Shallow interspecific divergence with *Praomys tullbergi* |
| GQ121052 *Pipanacoctomys aureus* | Shallow interspecific divergence with *Octomys mimax* |
| *Ctenomys lami*  *Ctenomys minutus* | Shallow interspecific divergence with each other |
| HQ693908, HQ693907 *Chaetodipus arenarius* | Shallow interspecific divergence with *Chaetodipus dalquesti* |
| EF156848 *Heteromys anomalus* | deep intraspecific divergence |
| JF459227 *Phyllostomus elongatus* | deep intraspecific divergence |
| JF444069 *Rhinolophus pusillus* | Shallow interspecific divergence with *Rhinolophus lepidus* |
| JF446032 *Lonchorhina aurita* | Shallow interspecific divergence with *Micronycteris megalotis* |
| JF443987 *Myotis muricola* | Shallow interspecific divergence with *Myotis horsfieldii* |
| HQ689950 *Pteropus samoensis* | Shallow interspecific divergence with *Myotis volans* |
| JF448053 *Lonchophylla thomasi* | deep intraspecific divergence |
| JF459227 *Phyllostomus elongatus* | deep intraspecific divergence |
| HQ919729 *Sturnira lilium* | Shallow interspecific divergence with *Sturnira tildae* |
| JF459166 *Glossophaga soricina* | Deep intraspecific divergence |
| JF459228 *Phyllostomus hastatus* | Deep intraspecific divergence |
| HM541798, HM541797, HM541796, HM541799, HM541801, HM541800 *Rhinolophus siamensis* | Shallow interspecific divergence with *Rhinolophus macrotis* |
| JF444044, JF444047 *Rhinolophus cf. lepidus* | Shallow interspecific divergence with *Rhinolophus cf. pusillus* |
| JF444098 *Rhinolophus shameli* | Shallow interspecific divergence with *Rhinolophus chaseni* |
| JF444099, JF444100 *Rhinolophus shameli* | Shallow interspecific divergence with *Rhinolophus stheno* |
| HM914945, HM914955, HM914956 *Hipposideros grandis* | Shallow interspecific divergence with *Hipposideros larvatus* |
| HM540204, HM540205 *Cynopterus horsfieldii*  HM540202, HM540203 *Cynopterus brachyotis* | Shallow interspecific divergence with each other |
| JF442389, JF442390 *Epomophorus labiatus* | Shallow interspecific divergence with *Epomophorus gambianus* |
| JF443981 *Myotis muricola*  HM540286 *Harpiola isodon* | Shallow interspecific divergence with each other |
| JN311814 *Peropteryx macrotis* | Shallow interspecific divergence with *Cormura brevirostris* |
| JF459204 *Molossops temminckii* | Shallow interspecific divergence with *Molossops neglectus* |
| HM914921 *Tylonycteris robustula* | Shallow interspecific divergence with *Tylonycteris pachypus* |
| GU723154 *Lasiurus seminolus*  GU723151 *Lasiurus borealis* | Shallow interspecific divergence with each other |
| GU72321 *Myotis ciliolabrum* | Shallow interspecific divergence with *Myotis californicus* |
| GU723209 *Myotis californicus*  GU723210 *Myotis ciliolabrum* | Shallow interspecific divergence with each other |
| JX008074 *Myotis nipalensis* | Shallow interspecific divergence with *Myotis aurascens* |
| *Myotis muricola*  *Myotis ater* | Shallow interspecific divergence with each other |
| *Artibeus cinereus*  *Artibeus glaucus* | Shallow interspecific divergence with each other |
| JF459220 *Phyllostomus discolor* | Shallow interspecific divergence with *Artibeus cinereus* |
| JF459067 *Artibeus cinereus* | deep intraspecific divergence |
| JF459487 *Pteronotus gymnonotus* | Shallow interspecific divergence with *Pteronotus davyi* |
| JX008071 *Myotis mystacinus* | deep intraspecific divergence |
| *Myotis myotis*  *Myotis blythii* | Shallow interspecific divergence with each other |
| JF444936 *Molossus rufus* | deep intraspecific divergence |
| FR856759 *Nyctalus leisleri* | Shallow interspecific divergence with *Nyctalus noctula* |
| JX008080 *Pipistrellus kuhlii* | Shallow interspecific divergence with *Pipistrellus pipistrellus* |
| HM102310 *Pongo pygmaeus* | Shallow interspecific divergence with *Pongo abelii* |
| HQ622760 *Hylobates agilis* | Shallow interspecific divergence with *Hylobates muelleri* |
| AY972708 *Hylobates agilis* | Shallow interspecific divergence with *Hylobates lar* |
| JQ821838, NC_018059 *Rhinopithecus strykeri* | Shallow interspecific divergence with *Rhinopithecus bieti* |
| AY972692 *Colobus polykomos*  EU185734 *Colobus guereza* | Shallow interspecific divergence with each other |
| AY671790 *Cercopithecus cephus* | deep intraspecific divergence |
| EF597500, NC_009747, EF597501 *Chlorocebus pygerythrus* | Shallow interspecific divergence with *Chlorocebus aethiops* |
| EF597503, NC_008066 *Chlorocebus sabaeus* | Shallow interspecific divergence with *Chlorocebus aethiops* |
| *Papio anubis*  *Papio cynocephalus*  *Papio hamadryas* | Shallow interspecific divergence with each other |
| *Saimiri sciureus*  *Saimiri boliviensis* | Shallow interspecific divergence with each other |
| FJ785421 *Aotus lemurinus*  AY250707, HM102288 *Aotus trivirgatus* | Shallow interspecific divergence with each other |
| GQ259899 *Nycticebus bengalensis*  NC_002765, AJ309867 *Nycticebus coucang* | Shallow interspecific divergence with each other |
| GQ259900 *Nycticebus coucang*  GQ259903 *Nycticebus javanicus* | Shallow interspecific divergence with each other |
| AY671787 *Otolemur garnettii* | Shallow interspecific divergence with *Otolemur crassicaudatus* |
| AY972682 *Galago moholi* | Shallow interspecific divergence with *Galago senegalensis* |
| Lepus mandshuricus  Lepus timidus  Lepus capensis | Shallow interspecific divergence with each other |
| JQ601063 *Sylvilagus floridanus* | deep intraspecific divergence |
| DQ347427 *Ochotona alpina* | deep intraspecific divergence |
