# Supplementary material for: Assessing DNA Barcoding as a Tool for Species Identification and Data Quality Control
Source: PLoS One. 2013 Feb 19;8(2):e57125. doi: 10.1371/journal.pone.0057125 (PMC3576373; doi:10.1371/journal.pone.0057125)
Supplement: Table S2 — Potential error ratio for CoxI sequences in mammals. (DOCX) [file pone.0057125.s003.docx]

| Groups | The number of raw sequences (before trimming) | The number of sequences (after trimming) | The number of potential error sequences | Error ratio |
| --- | --- | --- | --- | --- |
| primates | 17698 | 803^a^ | 17 | 2.12% |
| bats | 15490 | 15354 | 43 | 0.28% |
| rodents | 8955 | 8742 | 33 | 0.38% |
| even-toed ungulates | 1835 | 1789 | 30 | 1.68% |
| insectivores | 1261 | 1259 | 4 | 0.32% |
| carnivores | 1191 | 331 | 1 | 0.30% |
| rabbits | 216 | 215 | 2 | 0.93% |
| odd-toed ungulates | 205 | 204 | 1 | 0.49% |
| marsupials | 520 | 510 | 1 | 0.20% |
| othes | 146 | 146 | 2 | 1.37% |

Note: ^a^ exclude the human sequences.
